# Carcinoma of the Tongue

**Published:** 1882-05

**Authors:** 


Operation:

October 20, patient was anæsthetized, and trials were made at straightening the foot, but failed. Accordingly, on the 26th, they proceeded to cut. They removed a piece of the malleolus with a chain saw, resecting a piece of the bone, and bringing the remaining pieces together, thus straightening the foot. The wound was dressed with iodoform, and covered with an Argantin bandage.

Course:

### CARCINOMA OF THE TONGUE.

A. Uiberwassir, a peasant forty-nine years of age, was admitted November 30, 1881. He had been complaining for the past four months of a stinging pain in the left half of the tongue; three weeks later, he noticed an induration, which grew steadily, but caused no pain, except in eating. Lately, the tongue ulcerated, and another painless tumor appeared eight days later, behind the left lower maxillary bone. Patient had never contracted syphilis, or any venereal affection. There was no history of hereditary diseases in the family, and he never smoked.

Present condition:

The patient was of a medium stature, well built, well fed, and otherwise normal. On the left side of the tongue, between the middle and the anterior third, there was a fungiform papilla, whoae surface was covered with lardaceous granulations. The tumor had grown as far as, but had not affected, the glosso-palatal arch. It was impossible to ascertain how deep the tumor extended into the tongue, but the infiltration had not reached beyond the middle line. It had grown beneath into the maxilla, and was hard, though not painful. Patient had a fetid breath. Another tumor was found behind the condyle of the lower jaw. It was the size of a hazel nut, was movable, painless, and did not adhere to the skin.

*Treatment.*—Iodoform tampons were introduced into the mouth, after which the fœtor ceased, and the ulcerations healed.

Operation:

November 9, the patient was anæsthetized, the parts carefully cleansed, a circular incision was made over the lymphatic tumors, scraping the lower maxillary bone, and the lingual artery dissected out. The submaxillary glands, which were enlarged, though otherwise normal, were removed. The external maxillary artery was tied, as well as the lingual, seven or eight diseased lymph glands were removed, and it was found that the tumor extended to the external carotid artery, and included the internal jugular vein, so that a portion of the latter, two cent. in length, was removed between two ligatures. Iodoform gauze was applied. A speculum was introduced into the mouth, and the tongue drawn out with a hook. The tumor was now cut out with scissors, and a few remaining pieces were removed later. Half of the tongue was thus removed. There had been but little hæmorrhage, and it had been stopped with tampons of iodoform gauze. A large drainage tube was laid in the internal wound, and a smaller one laid in the external wound, iodoform dressings were applied, and covered over with oil silk waterproof cloth (McIntosh) and bandages. Patient was fed through a tube.

Course:

November 10, the temperature was 38 C. Patient was able to drink without difficulty. The dressings were removed, the drainage tube cleansed, iodoform re-applied, and dressings renewed.

November 11, no fever; patient speaks intelligibly, and drinks without pain. A fibrinous exudation had taken place in the pharynx on the edge of the wound, and it was insufflated with iodoform. The discharge had diminished, and the general condition was good.

November 13, both drainage tubes were taken away. November 15, the tampons were renewed. The discharge had no bad odor. November 16, Lister's dressings were removed. The external wound had united, except at one point, where some discharge oozed. No more tampons had to be introduced into the mouth.

November 21, complete healing of all the wounds; patient speaks pretty well, and eats almost anything without any difficulty.

---

EXCISION OF THE PYLORUS.—At the meeting of the Royal Medical Society of Vienna, on December 16, Dr. Wölfler exhibited a woman upon whom Professor Billroth had performed resection of cancerous pylorus two months before. The procedure in that case differed from that of others, in that the duodenum was not at once divided, but its posterior wall was first fixed to the stomach by sutures. The operation lasted an hour and a half. Microscopic examination showed that glandular cancer existed, but that no cancer elements had yet appeared in the lymphatic glands extirpated at the same time. Dr. Wölfler also showed the woman upon whom he had similarly operated eight months before. No recurrence had taken place. Her digestion is quite normal. —*Lancet.*

SMALL-POX, as an epidemic, now seems to be retiring before the well-directed efforts of local and general sanitarians. It has made its appearance, so far, at only five localities in Tennessee, viz.: Martin, Memphis and Fayette counties, in the western; Kingston in the middle, and Galveston in the eastern division of the State. The one case at Kingston (only eighteen miles off, and the nearest to Nashville) has had none to follow in its train. It was contracted from exposure to contagion on a steamer from Pittsburg. The case recovered. So far, Nashville has not had a single case for now six years. So much for thorough vaccination.—*Southern Practitioner.*

